# Updating the role of obesity and cholesterol in breast cancer

**DOI:** 10.1186/s13058-019-1124-1

**Published:** 2019-03-01

**Authors:** Laura Garcia-Estevez, Gema Moreno-Bueno

**Affiliations:** 1 Breast Cancer Department, MD Anderson Cancer Center, Arturo Soria 270, 280339 Madrid, Spain; 2 Biochemistry Department, Universidad Autónoma de Madrid (UAM), Instituto de Investigaciones Biomédicas ‘Alberto Sols’ (CSIC-UAM), IdiPaz, & Centro de Investigación Biomédica en Red de Cáncer (CIBERONC), Madrid, Spain

**Keywords:** Lifestyle factors, White adipose tissue, Inflammation, Estrogen, Progesterone, Menopause

## Abstract

**Background:**

Breast cancer is the second most common cause of cancer-related death among women. Advances in our understanding of the disease have translated into better diagnostics and more effective therapeutics, leading to earlier detection and improved outcomes. Several studies have pointed at lifestyle and environmental factors as contributory for the onset and progression of the disease. Obesity and cholesterol stand out for their potential causal relationship with breast cancer and ease of modification.

**Main text:**

Obesity and cholesterol represent risk factors for breast cancer, but their impact is largely affected by cofounding variables including menopausal status, disease subtype, and inflammation. Establishing a causal relationship between lifestyle factors and clinical outcomes may be challenging. Epidemiological studies and meta-analyses have rendered conflicting or sometimes contradictory results, possibly owing to the multifactorial nature of the disease. We discuss the supporting evidence and limitations in our understanding of obesity and cholesterol as risk factors for breast cancer.

**Conclusions:**

There is sufficient evidence that obesity and cholesterol impact clinical outcomes. Physicians are advised to take steps to help patients with their weight, such as recommending dietary and lifestyle interventions.

## Background

Breast cancer is the most prevalent cancer in women and the second leading cause of cancer death worldwide. In the European Union, an estimated 358.967 new cases and 90.665 breast cancer-related deaths are reported every year [1].

The classification of breast carcinoma relies on clinicopathological features and the expression of estrogen receptor (ER), progesterone receptor (PR), and human epidermal growth factor receptor 2 (HER2). Tumors expressing hormonal receptors constitute the most common breast cancer subtype, accounting for 60–70% of cases.

Genetic profiling, age of menarche and menopause, parity, age of first child, previous occurrence of cancer, and lifestyle are well-known risk factors for breast cancer. Although the identification of hereditable genetic factors has been critical for our understanding of the disease—and invaluable for providing women with the choice of preventative resection surgery—*BRCA1/2* mutations account for a small percentage (5–10%) of cases [2].

Lifestyle is considered an increasingly important contributing factor to breast cancer etiology. Obesity, overweight, metabolic syndrome, alcohol and hypercholesterolemia represent risk factors for breast cancer, whereas regular exercise appears to be protective [3]. However, their role in breast cancer remains largely unknown. Identifying lifestyle factors and understanding their effector mechanisms is paramount for establishing new primary prevention rules for breast cancer.

### Obesity, overweight, and breast cancer risk

Obesity, defined as a body mass index (BMI) of ≥ 30 kg/m2, affects over 600 million adults worldwide. The World Health Organization estimates that 40% of adult women are overweight, with prevalence tripling between 1975 and 2016 [4].

The impact of obesity on breast cancer risk differs across menopausal status and disease subtypes. Current evidence suggests that a high BMI associates with a reduced risk of premenopausal breast cancer, but strongly correlates with an increased risk after menopause [5]. Two meta-analyses on premenopausal women with ER+ breast cancer showed an inverse association between BMI and ER-positivity [6, 7]; obesity was more frequent in patients with ER−/PR− than with ER+/PR+ tumors (OR, 1.49; 95%CI, 1.29–1.73; *p* = 1 × 10^−7^). This observation is corroborated by most studies, indicating that obesity associates with a higher risk of breast cancer in premenopausal ER-negative and triple-negative breast cancers (TNBC) [6, 8].

Postmenopausal obesity seems to be a risk factor for the onset of hormone receptor-positive breast cancer in postmenopausal women [7, 9–11]. The relationship between BMI and postmenopausal breast cancer appears to be limited to ER+/PR+ (RR, 1.39; 95%CI, 1.14–1.70) but not ER−/PR− (RR, 0.98; 95%CI, 0.78–1.22) breast cancer [7]. A Women’s Health Initiative trial showed that BMI associated with a higher risk of ER+ and PR+ breast cancer, with hazard ratios (HR) increasing with each BMI level (HR, 1.86; 95%CI, 1.60–2.17 for BMI ≥ 35.0) [11]. The Million Women Study in the UK identified a nearly 30% higher risk of developing postmenopausal breast cancer with obesity (RR, 1.29; 95%CI, 1.22–1.36) [12]. Likewise, a meta-analysis of 34 studies comprising > 2.5 million women showed that postmenopausal breast cancer risk positively associates with each 5 kg/m2 increase in BMI (RR, 1.12; 95%CI, 1.08–1.16 [*p* < 0.0001]) [13].

An additional concern is the role of weight change across the life course. In this context, a prospective observational study of 74,177 women by Rosner and colleagues [14] indicated that weight loss > 5 kg since age 18 was inversely associated with risk of postmenopausal breast cancer, whereas weight gain after that age correlated with an increased risk—and the timing of weight gain either before or after menopause did not modify the risk.

### Obesity and breast cancer prognosis

Obesity and overweight have been linked with shorter all-cause and breast cancer survival. Ewertz and colleagues determined that the risk of developing distant metastasis in early-stage breast cancer patients (including ER+, ER− and unknown tumors) with a BMI ≥ 30 kg/m2 increased by 42 to 46% after 10 years, compared to patients with a BMI < 25 kg/m^2^, and the risk of death due to breast cancer after 30 years was significantly increased by 38% in patients with a BMI ≥ 30 kg/m2 [15, 16]. A separate study in TNBC patients failed to unequivocally demonstrate an association between obesity and mortality, as opposed to ER-positive women whose risk of death was threefold higher [17]. Furthermore, a meta-analysis of 43 studies reported a HR of 1.33 (95%CI, 1.19–1.50) for breast cancer-related mortality when contrasting obese and non-obese breast cancer patients [18].

The largest meta-analysis to date on this topic was conducted by Chan et al. comprising 213,075 patients from 82 studies [19]. The authors compared total and breast cancer-specific mortality in obese, overweight, normal-weight, and under-weight patients, before and after diagnosis. Before diagnosis, the summary relative risk (RR) for total mortality and breast cancer-specific mortality were 1.41 (95%CI, 1.29–1.53) and 1.35 (95%CI, 1.24–1.47), respectively, for obese vs. normal-weight patients. This positive association remained when BMI was measured within and after 12 months from diagnosis, regardless of menopausal status. The RR for total mortality and breast cancer-specific mortality for overweight vs. normal-weight patients at baseline were lower (1.07 [95%CI, 1.02–1.12] and 1.11 [95%CI, 1.06–1.17], respectively), but remained statistically significant [19]. Similar summary risk estimates were obtained when adjusting for confounding factors including tumor stage.

Despite an apparent causal relationship between BMI and survival, data is conflicting when considering breast cancer subtypes. A meta-analysis of 21 studies, analyzing the association between obesity, hormone receptor, and menopausal status [20], reported increased pooled HR for OS in heavier vs. lighter women both in ER+/PR+ and ER−/PR− cancers and in pre- and postmenopausal women groups. However, differences between groups lacked significance (*p* = 0.31 and *p* = 0.57, respectively), implying that the impact of obesity on breast cancer outcome is independent of hormone receptor or menopausal status. Clearly, prospective studies are required to further elucidate the role of obesity in different disease groups.

A summary of clinical studies investigating the association of obesity with breast cancer is presented in Table 1.

**Table 1:** Summary of studies investigating the association of obesity with breast cancer

Reference	Study type	Treatment	Results	Measure of association
Yang 2011 [6]	Meta-analysis	n.a.	Obesity in women ≤ 50 years is more frequent in ER(−)/PR(−) tumors Obesity in women > 50 years is less frequent in ER(−) tumors	*p* = 1 × 10^–7^ *p* = 6 × 10^–4^
Munsell 2014 [7]	Meta-analysis	Estrogen-progestin	Obesity associates with risk of hormone receptor-positive breast cancer: Premenopausal Postmenopausal	RR, 0.78; 95%CI, 0.67–0.92 RR, 1.39; 95%CI, 1.14–1.70
Pierobom 2013 [8]	Meta-analysis	n.a.	Obesity associates with TNBC tumors in premenopausal women	OR, 1.43; 95%CI, 1.23–1.65
Enger 2000 [62]	Case-case/case-control	n.a.	Obesity associates with ER(+)/PR(+) in postmenopausal women	OR, 2.45; 95%CI, 1.73–3.47
Rosenberg 2006 [63]	Population-based	Estrogen alone Estrogen-progestin	Weight gain > 30 kg in adulthood associates with risk of ER(+)/PR(+) tumors	OR, 1.5; 95%CI, 1.2–1.8
Nagrani 2016 [9]	Case-control	HRT patients excluded	Premenopausal women with a BMI ≥ 30 are at lower risk of breast cancer Women postmenopausal for ≥ 10 years are at higher risk of breast cancer	OR, 0.5; 95%CI, 0.4–0.8 OR, 1.8; 95%CI, 1.1–3.3
Suzuki 2006 [64]	Population-based	Oral contraceptives Postmenopausal hormones	Obesity associates with risk of developing ER(+)/ER(+) tumors in postmenopausal women	RR, 1.67, 95%CI, 1.34–2.07
Ahn 2007 [10]	Prospective	Postmenopausal hormones	Weight gain after age 18 associates with postmenopausal breast cancer risk in menopausal hormone therapy non-users	RR, 2.15, 95%CI, 1.35–3.42
Neuhouser 2015 [11]	Randomized	Postmenopausal hormones	Obesity and overweight associate with increased risk of invasive breast cancer 5% body weight gain in women with BMI < 25 associates with increased breast cancer risk	HR, 1.58; 95%CI, 1.40–1.79 HR, 1.36; 95%CI, 1.1–1.65
Reeves 2007 [12]	Prospective	HRT	Increasing BMI associates with increasing incidence of breast cancer in postmenopausal women	RR, 1.40; 95%CI, 1.31–1.49

*n.a* not available, *HRT* hormone replacement therapy

### Obesity, chronic inflammation, and breast cancer

Excessive caloric intake or reduced caloric expenditure leads to expansion of adipose compartments via hyperplasia and/or adipocyte hypertrophy. These conditions alter the physiology of white adipose tissue (WAT), causing dysregulation in the production of steroid hormones and adipokines, and chronic subclinical inflammation. Such alterations have been linked to carcinogenesis, tumor progression, and metastasis [21].

**Fig. 1:**
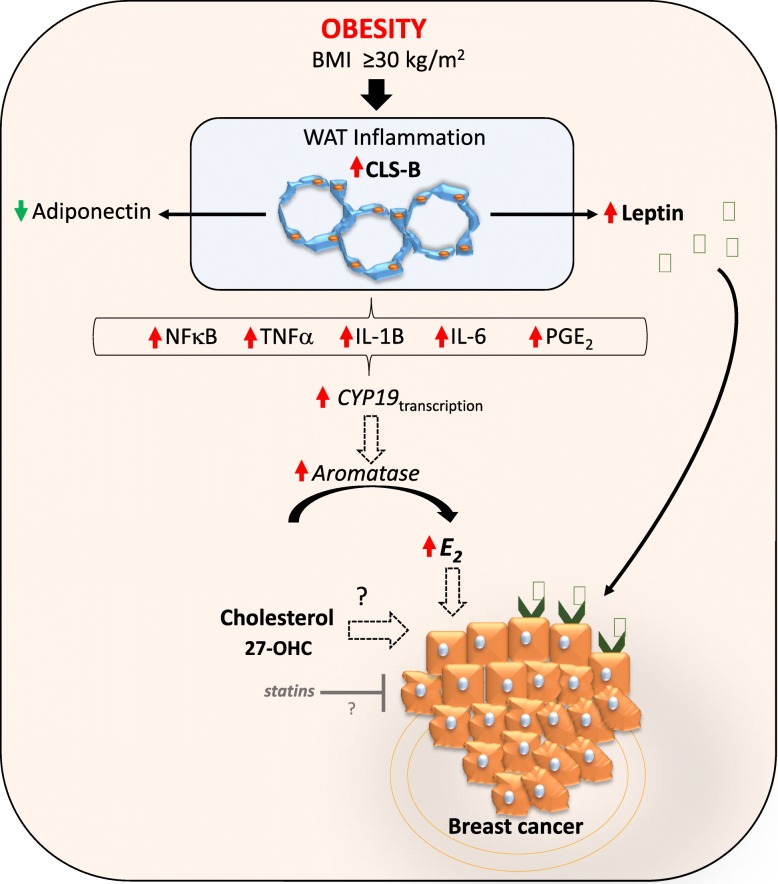
Schematic representation of the molecular relationship between obesity, inflammation, cholesterol, and breast cancer. NFkB—nuclear factor kappa-light-chain-enhancer of activated B cells; TNFα—tumor necrosis factor alpha; IL—interleukin; PGE2—prostaglandin E2; WAT—white adipose tissue; CLS—crown-like structures; 27-OHC—27 hydroxycholesterol; E2—estradiol

Adipose tissue inflammation may represent the physiological link between obesity and breast cancer. Inflamed adipose tissue is characterized by infiltrating macrophages encircling dead or dying adipocytes in a configuration termed crown-like structures (CLS) [22]. The presence of CLS in breast adipose tissue (CLS-B) is associated with activation of NF-κB and increased levels of pro-inflammation factors, resulting in upregulation of estradiol (E2). The upshot is that locally produced estrogens as a result of obesity-related WAT inflammation-axis might represent key drivers for hormone-dependent breast cancer development in postmenopausal women (Fig. 1).

Iyengar and collaborators provided the first evidence that postmenopausal women have larger adipocytes and greater prevalence and severity of WAT inflammation in the breast [23]. Strikingly, 90% of obese women had CLS-B. Breast WAT inflammation at diagnosis is associated with a 6-month shorter distant relapse-free survival (dRFS) in women who developed metastatic disease; results remained significant when adjusting for prognostic factors including BMI.

Adipocytes produce adiponectin and leptin, which participate in the regulation of caloric intake and metabolism, inflammation, angiogenesis, and cell proliferation. Breast cancer cells are surrounded and influenced by this microenvironment. Leptin appears to be strongly involved in mammary carcinogenesis and may contribute to the local pro-inflammatory mechanisms, especially in obese patients. There is a positive correlation between BMI index and leptin levels, whereas adiponectin concentrations generally decrease with greater adiposity (Fig. 1). The increased leptin-adiponectin ratio seen in obesity has been implicated in neoplastic transformation and tumor progression [24].

Leptin may act as a molecular link between obesity and breast cancer [25]. Breast cancer cells overexpress leptin receptor, hence becoming highly susceptible to the influence of elevated leptin levels typically seen in obese patients [26]. Leptin displays pleiotropic effects that include inhibition of pro-apoptotic signals in breast cancer cells, sensitization to estrogens, and modulation of the tumor microenvironment, contributing to local pro-inflammatory mechanisms and promoting mammary tumor growth (Fig. 1) [27, 28]. Increased leptin levels in breast cancer patients have been linked with risk of metastasis and reduced survival [26]. Niu and collaborators conducted a meta-analysis including pre- and postmenopausal breast cancer women and healthy controls, as well as lymph node metastasis positive cases, and concluded that leptin levels play a role in breast cancer [29].

### Cholesterol and breast cancer risk

High blood cholesterol is a common comorbidity in obesity [30]. Its impact as a risk factor for breast cancer is conflicting, and it is unclear whether total, LDL, or HDL cholesterol contribute to the disease [31]. It is important to differentiate the association between total cholesterol and breast cancer risk, and the role of high-fat diet for disease onset. Moreover caution is needed when interpreting results, as blood cholesterol levels may be influenced by different cancer mechanisms [32].

Touvier and collaborators conducted the first systematic review and meta-analysis of prospective studies investigating the association between total cholesterol (TC), HDL-C, LDL-C, ApoA1, and ApoB with breast cancer risk [33]. Their analysis suggested a modest but statistically significant inverse association between pre-diagnostic total cholesterol levels (dose–response HR, 0·94; 95%CI 0·89–0·99, seven studies, *I*^2^ = 78%; highest vs. lowest HR, 0·82; 95%CI 0·66–1·02; nine studies, *I*^2^ = 81%) and HDL-C levels (dose–response HR, 0·81; 95%CI 0·65–1·02, five studies, *I*^2^ = 30%; highest vs. lowest HR, 0·82; 95%CI 0·69–0·98, five studies, *I*^2^ = 0%) with breast cancer risk [33].

A recent prospective study investigating the association between pre-diagnostic serum lipid concentrations and breast cancer risk and survival reported that, overall, serum lipids did associate with breast cancer risk, regardless of BMI and tumor ER status or when accounting for time between blood collection and diagnosis [34]. By contrast, a large study in over 664,000 women utilizing Big Data from the UK Algorithm for Co-morbidity, Associations, Length of Stay and Mortality (ACALM) registry found an association between hyperlipidemia and breast cancer. The ACALM study demonstrated that women above 40 with high cholesterol were 45% less likely to develop breast cancer than those without high cholesterol [35]. Of the patients who developed breast cancer, those with high cholesterol had a 40% lower risk of death [35]. The authors concluded that women with high cholesterol have strikingly lower rates of breast cancer and improved survival outcomes. One potential explanation for these findings is that the use of statins to lower cholesterol levels may reduce the risk of breast cancer, breast cancer recurrence, and mortality rates [36]. However, evidence from several meta-analyses for the protective effect of statins in breast cancer is nowadays conflicting [37, 38].

A large prospective trial of postmenopausal women evaluated the association between serum total cholesterol and breast cancer risk taking BMI into account [39]. In an age-adjusted model, there was a positive association between cholesterol levels and breast cancer (p-trend, 0.0024); however, the association lost significance when adjusting for BMI (p-trend, 0.0684).

### 27-Hydroxycholesterol and breast cancer

27-OHC ((3β,25R)-Cholest-5-ene-3,26-217diol) is a primary metabolite of cholesterol hydroxylation mediated by CYP27A1 and circulates at comparable or slightly higher concentrations compared to other oxyteroles [40].

In models of cardiovascular disease, 27-OHC behaves as an ER antagonist, while in osteoblasts and cellular models of ER-positive breast cancer it functions as a partial ER agonist [41]. Based on these and other reports, 27-OHC is now considered an endogenously produced selective estrogen receptor modulator (SERM) [42].

SERMs modulate the activity of ER in a context-specific manner. Importantly, therapeutic use of SERMs significantly reduces the incidence of breast cancer [43]. The best characterized SERM is tamoxifen, which acts as an ER antagonist [44]. Intra-tumor levels of 27-OHC are sixfold higher in ER-positive breast tumors relative to the adjacent normal tissue [45] and act as a partial agonist in cell models of ER-positive breast cancer, stimulating their proliferation [46]. It has been hypothesized that 27-OHC rather than cholesterol per se is responsible for stimulating the proliferation of ER-positive breast cancer cells. Indeed, exogenous administration of 27-OHC promotes the growth of MCF7 xenografts, and the effect is reversed by co-treatment with ER antagonists [44].

27-OHC also has activity as a liver X receptor (LXR) agonist. LXR activation typically results in inhibition of cell proliferation secondary to cellular cholesterol efflux [47]. Co-activation of ER and LXR results in competitive intracellular signaling and cross-modulation, as demonstrated by loss-of-function in vitro experiments whereby 27-OHC activity on LXR-knocked-down cells enhanced the induction of ER target genes, whereas in ER-knocked-down cells resulted in significant up-regulation of LXR target genes [48]. In ER-positive breast cancer cells, the “ER-activity” of 27-OHC prevails over the growth inhibitory action of LXRs; however, the extent of these activities may be fine-tuned by cellular and microenvironmental cues impinging on either the LXR or ER axes.

Several studies have suggested an association between 27-OHC and serum cholesterol [49, 50]. Considering the role of 27-OHC in stimulating tumor growth in in vitro models of ER+ breast cancer, Lu and collaborators explored the association between 27HC and breast cancer risk in a nested case-control study, using the European Prospective Investigation into Cancer and Nutrition (EPIC)–Heidelberg cohort [51]. Although 27HC was not associated with breast cancer risk overall, the study identified that the association between 27HC levels and breast cancer risk differed by menopausal status at blood collection; whilst no relationship was observed among premenopausal women, postmenopausal women at blood collection had a statistically significant inverse association between 27HC levels and breast cancer risk. The authors considered that the “benefit” of 27HC-mediated inhibition of estradiol–ER binding outweighs the “harm” of the partial agonistic effect of 27HC in breast cancer. These results warranty additional experimental studies on the combined effects of estradiol and 27HC.

Few known breast cancer risk factors exhibit opposing effects in pre- and postmenopausal women. Apart from BMI, which has been covered in the previous section, and of circulating 27HC levels as reported above, further studies in well-characterized populations, including pre- and postmenopausal women at diagnosis and at blood collection, are required to confirm these findings.

Given the association between high cholesterol and worse prognosis, and of statin usage with shorter recurrence-free survival [52], there is significant interest in the potential benefit of cholesterol-lowering medication in breast cancer patients. In the Breast International Group 1–98 study [53], patients initiating cholesterol-lowering medication in combination with standard endocrine therapy demonstrated longer disease-free survival, breast cancer-free interval, and distant recurrence-free interval.

### Dietary cholesterol intake/fat intake and breast cancer risk

Generally, dietary saturated fat intake is synonym of dietary cholesterol intake. It is well established that saturated fat raises low-density lipoprotein (LDL) cholesterol, a leading cause of atherosclerosis and cardiovascular disease [54]. Saturated fat is found in animal foods such as red meat, poultry, and full- or reduced-fat dairy products.

Li and collaborators conducted the first meta-analysis examining the association between dietary cholesterol and breast cancer [55]. The pooled RR for the highest (median 394 mg/day) vs. the lowest (median 138 mg/day) dietary cholesterol categories was 1.29 (1.06–1.56). For dose–response analysis, a nonlinear association was found between dietary cholesterol and breast cancer; and the association became significant when the cholesterol consumption was greater than 370 mg/day.

These results strongly suggest an association of dietary cholesterol and breast cancer. By contrast, some epidemiological studies have failed to reach similar conclusions or have restricted the impact to specific groups of patients [56, 57] (Table 2).

**Table 2:** Summary of clinical studies investigating the association of cholesterol with breast cancer

Reference	Study type	Results	Measure of association*
Touvier 2015 [33]	Meta-analysis	Cholesterol associates with a reduced risk of breast cancer: total cholesterol (dose response) Total cholesterol (highest vs. lowest) HDL-C (dose–response) HDL-C (highest vs. lowest)	*HR, 0.94; 95%CI, 0.89–0.99* HR, 0.82; 95%CI, 0.66–1.02 HR, 0.81; 95%CI, 0.65–1.02 *HR 0.82; 95%CI, 0.69–0.98*
Bahl 2005 [52]	Prospective	Higher total cholesterol associates with a trend towards increased risk of recurrence	*p = 0.03*
Carter 2017 [35]	Retrospective	Hyperlipidaemia associates with a reduced risk of breast cancer	*OR, 0.67; 95%CI, 0.48–0.92*
Ha 2009 [39]	Retrospective	Positive association between cholesterol levels and breast cancer: In postmenopausal women (age-adjusted model) In postmenopausal women after adjusting for BMI	*p-trend = 0.0024* p-trend = 0.0684
Borgquist 2017 [53]	Phase III	Cholesterol-lowering medication associates with improved: Disease-free-survival Breast cancer-free interval Distant recurrence-free interval	*HR, 0.79; 95%CI, 0.66–0.95* *HR, 0.76; 95%CI, 0.60–0.97* *HR, 0.74; 95%CI, 0.56–0.97*
Li 2016 [55]	Meta-analysis	Dietary cholesterol associates with increased risk of breast cancer	*RR, 1.29; 95%CI, 1.06–1.56*
Hu 2012 [57]	Population-based	Association of high cholesterol intake with risk of breast cancer: Postmenopausal women Premenopausal women	*OR, 1.48; 95%CI, 1.07–2.07* OR, 1.10; 95%CI, 0.75–1.72
Undela 2012 [65]	Meta-analysis (observational studies)	Statin use does not significantly impact breast cancer risk Long-term statin use does not significantly impact breast cancer risk	RR, 0.99; 95%CI, 0.94–1.04 RR, 1.03; 95%CI, 0.96–1.11
Mansourian 2016 [66]	Meta-analysis (observational studies)	Statin use associates with reduced: Breast cancer recurrence Breast cancer mortality	*OR, 0.79; 95%CI, 0.73–0.85* *OR, 0.84; 95%CI, 0.82–0.87*
Islam 2017 [38]	Meta-analysis (observational studies)	Statin use does not associate with reduced breast cancer risk	RR, 0.94; 95%CI, 0.86–1.03

*Statistically significant associations are highlighted in italics

The Mediterranean diet is a good example of a low-fat diet. It is characterized by a high intake of virgin olive oil, vegetables, fruits, plant proteins, fish and other seafood, whole grains, nuts, and low-fat dairy, accompanied by moderate alcohol intake and low red meat consumption [58]. The beneficial effects of a Mediterranean diet have been noted with regards to decreasing the risk of breast cancer and breast cancer recurrence, while improving overall survival [59–61].

## Conclusion

Overweight and obesity are intimately related with breast cancer development and recurrence. The interplay between obesity, inflammation, and the tumor microenvironment drive tumor expansion, primarily in hormone-sensitive and postmenopausal patients. Lately, the role of leptin and leptin receptor in breast cancer cells has shed light on the role of the adipose environment in breast cancer.

Several meta-analyses have provided evidence that obesity carries a 35–40% increased risk of recurrence and death, irrespective of menopausal or hormone receptor status. In this regard, breast cancer prevention requires raising awareness among women for watching their body weight, especially those in menopause. This can be achieved through a low-cholesterol/low-saturated fat diet and regular exercise.

The role of total blood cholesterol for breast cancer development is controversial. Most studies show that high cholesterol levels prior to diagnosis protect against the development of tumors, prompting some authors to advocate the use of statins to lower cholesterol. It is uncertain that total cholesterol makes the most appropriate risk biomarker, with 27-OHC perhaps a more suitable candidate.

Collectively, clinical studies and meta-analyses support a role for obesity, dietary fat intake and/or cholesterol in the onset and progression of the disease, and practicing physicians should be pro-active in recommending habits such as an appropriate diet and regular physical activity to maintain a healthy weight.
